# Improving Viral Load Suppression Among Men and Children Active in Care Through Community-Designed and Led Solutions: Protocol for Retrospective Closed Cohort Study in Eastern Uganda

**DOI:** 10.2196/32784

**Published:** 2022-04-13

**Authors:** Krista J Odom, Amanda Ottosson, Joyce Draru, Harriet Komujuni, Esther Karungi Karamagi Nkolo, Taroub Harb Faramand

**Affiliations:** 1 WI-HER, LLC Vienna, VA United States

**Keywords:** HIV/AIDS, viral load suppression, Uganda, people living with HIV, 95-95-95, social and behavior change, USAID, gender, youth, and social inclusion, virus, HIV, AIDS, antiretroviral therapy, behavioral science, implementation science, behavior change, men, children, community design, methodology

## Abstract

**Background:**

In collaboration with facilities, communities, district local government, and the United States Agency for International Development (USAID) implementing partners, the iDARE methodology was implemented at the community level to address root causes of low HIV antiretroviral therapy adherence among men and children actively enrolled in care, resulting in low viral load suppression (VLS) in two districts in the eastern region of Uganda. The methodology encourages the use of cocreated sustainable solutions addressing gender, youth, and social inclusion issues to reduce barriers to care and reach the 95-95-95 Joint United Nations Programme on HIV/AIDS target for HIV epidemic control. We aim to measure the impact of iDARE on VLS for men and children active in care and investigate the practical scale up of the solutions designed using the iDARE methodology.

**Objective:**

The primary objective of this study will be to measure the implementation impact of the iDARE methodology at the facility and community levels on VLS for people living with HIV. The secondary objective is to investigate the practical scale up of the iDARE methodology using evidence-based gender, youth, and social inclusion social behavior change packages to rapidly meet the Ugandan Ministry of Health targets for VLS.

**Methods:**

A retrospective cohort study design will be used to analyze program data that aims to increase the rates of VLS in men and children who are classified as active in care using community engagement and quality improvement techniques. We will examine 3 pilot health centers’ data from a USAID-funded program aimed at social behavior change to increase health-seeking behavior in Uganda. Based on the iDARE process and results, change packages were developed to highlight lessons learned and best practices in order to share with subsequent implementation sites.

**Results:**

The USAID-funded Social and Behavior Change Activity began implementation of iDARE in September 2020, with baseline data collected in August 2020.

**Conclusions:**

Data on viral load suppression was collected from facilities on a monthly basis to record progress toward the 95-95-95 goal. The expected primary outcome is an increase in actively enrolled men and children reaching VLS in order to meet the Ugandan Ministry of Health target of 95% VLS among those active in care.

**International Registered Report Identifier (IRRID):**

DERR1-10.2196/32784

## Introduction

### Background

Despite the immense global effort, the HIV epidemic remains a threat and a leading cause of morbidity and mortality. Persistent disparities by geographic region and demographics, including age and sex, have led to an unbalanced burden of disease. Globally, there is a 10% difference in antiretroviral therapy (ART) rates between males and females, 68% and 79%, respectively [1]. There are also regional disparities, especially in Sub-Saharan Africa (SSA) [2]. Globally, more than 37 million people are living with HIV, and two-thirds of those individuals are in SSA despite only having 15% of the global population [1,2]. Furthermore, in SSA, there is more than a 20% difference between adults and children classified as active in care for ART treatment, 74% of adults (aged ≥15 years) and 53% of children (aged <15 years) [1].

In Uganda, there are roughly 1.46 million people living with HIV, and HIV prevalence among adults aged 15-49 years is 6%, and among children aged 0-14 years, it is 0.5% [3]. While rates are dropping, HIV incidence continues to outpace morbidity; therefore, the overall prevalence of HIV in Uganda remains high [3,4]. Of the people living with HIV, 86% are enrolled in treatment and are active in care, meaning they are receiving ART [3]. Of those active in treatment, 89.8% have achieved viral load suppression (VLS) [3]. However, Uganda did not meet the 90-90-90 goals as set by the World Health Organization’s 2020 Global Health Sector Strategy on HIV, but the country is now working towards the new 95-95-95 goals to control the HIV epidemic. The 95-95-95 goal states that 95% of people living with HIV know their HIV status; 95% of people diagnosed with HIV receive ART, and 95% of people living with HIV on ART treatment achieve VLS [2,5]. Complicating the Ugandan response to the epidemic is the disparities in burden across geographical areas and between socio-economic and social demographic subpopulations.

In 2020, the United States Agency for International Development’s Social and Behavior Change Activity (USAID SBCA) in Uganda began with the vision of Uganda where individuals and communities are healthy, resilient, and supported by strong and adaptable systems and institutions to lead productive lives. SBCA works across Uganda in HIV, malaria, tuberculosis, gender-based violence, family planning, maternal newborn child health, and nutrition. Under this project, WI-HER began the application of the iDARE methodology to address gaps in VLS. iDARE, developed by Dr. Taroub Harb Faramand, was implemented to support the locally-led improvement of HIV VLS rates under the USAID SBCA program by focusing on gender, youth, and social inclusion (GYSI) gaps across subpopulations with a disproportionate burden of poor health outcomes. During a formative data review on HIV VLS in the eastern region of Uganda, WI-HER, in partnership with district local governments in Tororo and Kapachorwa districts, identified men and children as key subpopulations with the largest gap in VLS that was impacting district and overall national efforts in achieving 95-95-95 targets.

A detectable viral load in people living with HIV is associated with increased morbidity, mortality, and transmission [2]. Strong adherence to ART suppresses viral load to undetectable levels for people living with HIV, greatly reducing the risk of transmitting HIV to others. According to the Ugandan Ministry of Health (MOH) 2019 HIV Epidemiological Surveillance Report, VLS is 89.8% for people living with HIV who are on ART and 75% for all people living with HIV [3]. However, this is not consistent across age and sex-disaggregated data; 48% of children (aged <15 years) active in care for HIV have achieved VLS; for men, the rate is 68% [3]. In Uganda, low ART adherence for children and men has been associated with food insecurity, stigma, lack of caregivers for support, and forgetting [6-9].

As the third goal for achieving the 95-95-95 targets, VLS requires quality services from the facility but also adherence to treatment, including appointment keeping and clientele taking medication in a timely manner. In order to increase adherence, this requires the clientele to shift their behavior and/or mindset. For newly diagnosed individuals living with HIV, in addition to adjusting to life living with a chronic disease, GYSI factors impact an individual’s perceptions, beliefs, attitudes, and decisions, which impact their behavior and, ultimately, health outcomes, namely VLS. Social and behavior change (SBC) programs have a history of increasing VLS, especially among high-risk populations in Uganda [10,11]. SBC is rooted in Social and Behavior Theories that support the necessity of interventions aimed at improving health behaviors [12]. Community-based SBC interventions to improve the delivery of ART have been shown to reduce the disparities between males and females in VLS in SSA [13-15]. Additionally, SBC has been effective in increasing ART adherence for youth living with HIV in Uganda [16]. The Ugandan MOH and USAID SBCA have taken steps to streamline SBC efforts to increase VLS [10,11].

### Rationale

This study will provide practical evidence of the feasibility and efficacy of SBC to increase the rate of VLS within male and children subpopulations that bear a disproportionate burden of unsuppressed cases for those active in care. In addition to the efficacy of the iDARE methodology at improving VLS rates, the study will examine the feasibility and the scalability of the iDARE approach through the staggered timing of the clustered cohort design.

Evidence provided in this study will support the scaling of the iDARE methodology within Uganda, in partnership with the MOH, district local government, and implementing partners, to meet the 95-95-95 target set by the UNAIDS Global Health Sector Strategy on HIV. In addition to meeting the 95-95-95 target, improving adherence and increasing VLS will avoid the development of HIV antiretroviral drug resistance (HIVDR) in the Ugandan context. An early warning indicator assessment in Uganda indicated a high potential for HIVDR [3]. Raising numbers of HIVDR cases would lead to the need to shift ART strategies, which could result in cost increases for the MOH and international pharmaceutical support organizations. In the Ugandan 2019 HIV Epidemiological Surveillance Report, the MOH stated the need to improve the efficiency of HIV services to reduce morbidity and reduce the incidence of HIV so that Uganda may achieve control of the HIV epidemic [3].

### Aims

The primary aim of this study will be to examine the implementation impact at the facility and community levels of the iDARE methodology on VLS for people living with HIV. The secondary aim is to investigate the practical scale up of the iDARE methodology using evidence-based GYSI social behavior change packages to rapidly meet MOH targets of VLS.

## Methods

### Study Design

This is a retrospective closed cohort study reviewing program data collected directly from facilities from WI-HER SBC efforts through USAID SBCA. The cohorts are the pilot 3 facility catchment areas for implementation of iDARE to address VLS in men and children. iDARE implementation was staggered using lessons learned and best practices in the form of GYSI social behavior change packages from the preceding facilities to inform subsequent facilities. We hypothesize that the iDARE methodology will lead to a marked improvement in HIV VLS and will be rapidly scalable using GYSI social behavior change packages as guides. The actions taken and data collected were for the implementation of public health and clinical programs for those intended purposes.

### Ethical Considerations

Data analyzed for the retrospective study are public health program data; data were not collected for the purpose of research. Therefore, this study does not require IRB approval [17].

### Setting

Implementation of the iDARE methodology to increase VLS for men and children active in care took place at 3 pilot facilities across 2 regions in Uganda. Sites were selected based on accessibility, burden of disease, and local resources.

The Bukedi subregion, with a population of 2,296,686, is in the eastern region of Uganda at the border of Kenya [4,18]. Within the Bukedi subregion, implementation took place in Tororo District, with an estimated population of 607,803 [4,18]. The iDARE coach, along with the district health team, selected the Nagongera Health Center (HC) IV and Mulanda HC IV based on low levels of VLS among men and children for those classified as active in care at the baseline assessment using DHIS2 data in August and September 2020, respectively. Implementation of iDARE began in September of 2020 in the Nagongera HC IV and in October 2020 for Mulanda HC IV.

The Sebei subregion is also in the Eastern Region of Uganda, north of Bukedi subregion. Within the Sebei subregion, implementation took place in Kapachorwa District, which has an estimated population of 125,785 [4,18]. The iDARE coach, along with district health team and local implementing partners, selected Kabeywa HC III from a baseline assessment using DHIS2 data in December 2020, and implementation of iDARE to address low levels of VLS in men and children began in January 2021.

### iDARE

Grounded in behavior theory and human-centered design, the iDARE methodology is based on improvement science and drives locally-led solutions. The methodology, developed by Dr. Taroub Harb Faramand, draws from classic theories and concepts that have a robust evidence base and application in the field, such as the theory of planned behavior, the social cognitive theory, and the diffusion for innovations, as well as innovative approaches from parallel fields including behavioral insights, behavioral economics, and consumer/marketing research [19-21]. The iDARE methodology is a results-driven and inclusive methodology for supporting community-designed and led solutions and engagement to achieve SBC. It helps local stakeholders identify a shared goal, think through gaps, and together come up with sustainable, locally-owned solutions. iDARE promotes GYSI at its core to determine root causes of any gap or challenge and thinks through locally (contextually and culturally) appropriate ways to overcome the root causes and drive sustainable social and behavior change. The steps are as follows:

1. Identify challenges and/or gaps in achieving an overall goal or outcome with particular emphasis on identifying GYSI issues, gaps, or barriers.
2. Design effective local solutions for achieving the intended outcome alongside stakeholders and ensure inclusive participation from program constituents using a vast toolbox of best practices, resources, and methodologies.
3. Apply and assess activities with clear indicators and feedback loops for continuous improvement;
4. Record performance against indicators as well as successes, failures, and additional gaps and challenges that have arisen during implementation.
5. Expand and scale successful solutions to realize the greater impact and share activity experiences widely for learning.

Used as an implementation framework, iDARE emphasizes close partnerships with stakeholders and clear sustainability metrics for achieving an exit strategy to ensure that initiatives are locally-owned, sustainably delivered, responsibly managed, and properly documented. For the USAID SBCA program in Uganda, iDARE is used to bring together community and district-level stakeholders to support their communities to improve health outcomes.

### Social and Behavior Change Packages

Across the 3 pilot sites, implementation of iDARE was staggered to build upon lessons learned and best practices from the preliminary sites. Successful iDARE solutions were recorded into Social and Behavior Change Packages, categorized for the target populations, men and children. The change packages were then used in subsequent sites in order to rapidly share lessons learned.

iDARE teams use their iDARE journal to record existing and new gaps, barriers, and issues impacting their targeted goal/outcome. With the WI-HER coach’s support, iDARE teams transfer successful solutions recorded in the iDARE journal to develop the Social and Behavior Change Packages. These packages are a tool for iDARE teams to have a resource guide of successful solutions that led to social and or behavior change, with qualitative and quantitative data as evidence to justify the successful change. They indicate the clinical area of iDARE implementation, target population, GYSI barrier being addressed, solutions that have been implemented, including step-by-step guidance on how the solutions were implemented, and the results. These packages may then be used at subsequent sites to drive scale up and cross-site learning in order to direct iDARE teams towards useful solutions from other sites. While these solutions have worked in one site, it is important to note that iDARE is a process, and solutions must be tailored to the culture, resources, and priorities for every context.

### iDARE Teams

An iDARE coach supported the implementation of iDARE across all 3 sites. iDARE coaches are experts in SBC, GYSI, and improvement science trained by WI-HER on the iDARE methodology. iDARE implementation for improved VLS was supported by one iDARE coach across all 3 pilot sites. The iDARE coach across these 3 sites was directly trained by Dr. Faramand almost 10 years ago and has been working in the field using iDARE since.

The iDARE coach met with the district health teams and a local implementing partner to identify local facilities based on the epidemiological data for VLS within the Tororo and Kapachorwa districts. District health teams and local implementing partners were incorporated into the implementation process to build sustainable coaching and supervision within the local context after the exit of the iDARE coach. The district-level health team and local implementing partners make up the district-level iDARE teams.

The district-level iDARE team identified facility stakeholders for inclusion in the community-level iDARE teams through their direct role in HIV care and treatment or data management at the facility. For example, the lead nurse of the HIV clinic may be selected due to their direct interaction with HIV clients in the facility. District-level iDARE team members worked together with facility stakeholders to conduct semistructured interviews with nonsuppressed actively enrolled men and children (and their caregivers) to identify community influencers to add to the community-level iDARE team. Influencers were selected by the target populations themselves (in this case, the nonsuppressed actively enrolled men and children) through rapid analysis of interviews with existing target health facility patients and with the target populations at large. Examples of community influencers are priests, teachers, and local administration, but it is not necessary to have an official position to be a community influencer.

The facility team members and community influencers together make up the community-level iDARE teams. Across all 3 pilot sites, district and community-level iDARE teams were formed for the clinical health area of HIV VLS. At the community level, the iDARE coach worked with each community-level team to assign key roles, including community lead, clinical lead, and data focal person, based on the role of the team members within the facility and consensus from the wider community-level iDARE team.

**Figure 1:**
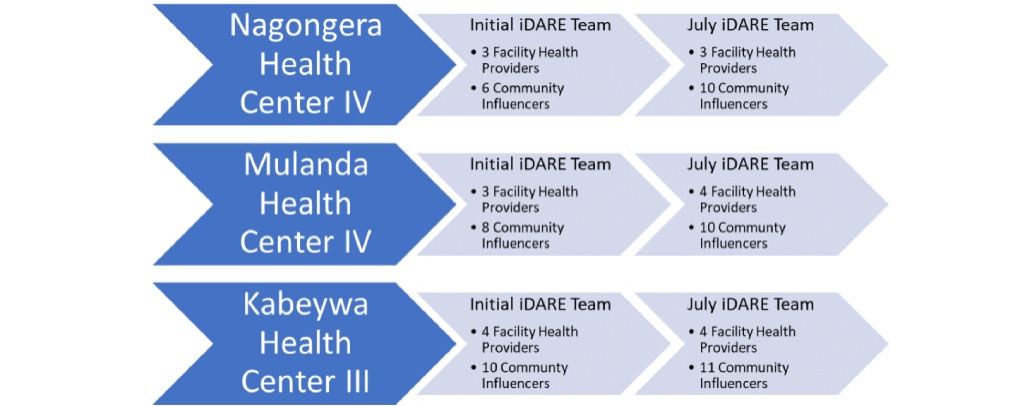
Community level iDARE teams from Nagongera, Mulanda, and Kabeywa Health Centers.

iDARE teams are flexible in their design and setup in order to rapidly address ongoing and/or newly identified gaps and challenges (Figure 1). At Nagongera HC IV, in the initial iDARE team, there were 3 facility health workers and 6 community influencers. However, during implementation, an additional 4 influencers were identified to join the team. In the initial Mulanda HC IV iDARE team, 3 facility health workers and 8 community influencers were included. During the iDARE process, an additional 2 influencers and 1 health worker were added to the team. At the Kabeywa HC III, 4 facility health workers and 10 community influencers were on the initial iDARE team, with an additional community influencer added since implementation.

### iDARE Implementation

The initial step in the implementation of iDARE at the community level is orientations facilitated by the iDARE coach with the community-level iDARE teams. With support from the coach, iDARE teams identify a SMART (specific, measurable, attainable, relevant, time-bound) goal and agree upon an indicator to measure progress in achieving the SMART goal. Orientations are followed by monthly check-ins between the iDARE coach, the district-level iDARE teams, and community-level iDARE teams to gather data and support the ongoing iDARE process to achieve the target goal. During these visits, additional coaching is done on topics of GYSI, data management, and continual root cause analysis. Continued coaching sessions are intended to support and build capacity in the iDARE teams, specifically in data management and continuous improvement in the iDARE team’s indicator. Within 6 months of iDARE implementation, in-person site visits drop from monthly to quarterly with monthly virtual data checks.

Facility orientations took place over 2 days and were conducted by the iDARE coach. The iDARE coach was consistent across all study sites. The focus of day 1 is GYSI concept sensitization and the introduction of the iDARE methodology. The second day focuses on the first 2 steps in the iDARE process. Namely, identifying root causes of low adherence to ART in the GYSI context. This is done through interviews and rapid analysis of people living with HIV and additional stakeholders within the community. Once the root causes of low adherence are identified, the community-level iDARE teams design SBC solutions to address the root causes. They then complete an action plan for implementation, including assigning roles and responsibilities within the iDARE team and developing indicators to measure success based on the clinical goal. For VLS, the facility used the 95-95-95 USAIDS goal; therefore, the indicator target was set to 95% VLS for those active in care. After the iDARE orientation, the solutions and action plans were applied by the responsible persons to address the GYSI gaps impacting health outcomes. The implementation process included the ongoing collection of the data to measure the impact of the solutions against the indicator target. In this process, the community-level iDARE teams assessed the solutions for the successes and failures. Data were recorded**,** along with processes, into the social and behavior change packages to share best practices and lessons learned with subsequent facilities that face similar GYSI gaps in VLS; in doing so, facilities can expand on the successful solutions across new implementation sites.

Due to the ongoing COVID-19 pandemic, lockdowns restricted movements between districts and led to adjustments to the iDARE implementation. During the first government lockdown in 2020 and the second in June to July 2021, monthly coaching visits were switched to a hybrid approach, and community-level iDARE teams came together at the facility for virtual support from the iDARE coach. Facilities practiced social distancing as well as mask and handwashing protocols to mitigate the risk of COVID-19.

Regular learning sessions were held to support the continuous learning and adaptation among iDARE teams within each site and across facilities. Through these meetings, lessons learned and best practices were shared; this process aided in the development of comprehensive Social and Behavior Change Packages for scaling up.

### Participants

There was no recruitment; all persons who qualified as having not achieved VLS despite being active in care at baseline were included from all 3 pilot catchment areas in closed cohorts. Recently diagnosed people living with HIV were encouraged to initiate ART. Six-months post-ART initiation, individuals were classified as active in care if they had been up taking ART at a 95% adherence and were eligible for viral load testing. Collecting data on all individuals active in care within the catchment greatly reduced sampling bias, as no samples were taken. This method also provided practical, real-world evidence for the implementation of iDARE at the community level.

Respectively, men and children active in care at the time of baseline were included in the study as distinct closed cohorts. All people living with HIV, active in care at the time of implementation within the iDARE catchment area, constitutes having been exposed to the iDARE intervention. Therefore, they are included in the population-level analysis.

### Outcomes

After an individual active in care reaches 95% adherence in the facility health records and maintains appointments for 6 months, they were then tested for VLS using serum and dried blood samples for a PCR test. Viral load suppression refers to the proportion of HIV RNA within the individual’s blood. To meet the criteria to be VLS, the individual must have HIV RNA <1000 copies per milliliter of plasma. The data was dichotomous, either confirmed as virally suppressed or not, and presented as a rate of the population level.

The primary outcome was the rate of VLS for those active in care at each of the 3 pilot facilities. The focal data person on the community-level iDARE team reviewed and reported from the facility records on people living with HIV that were active in care. Facility HIV registries were the source document; they hold de-identified client records on HIV, ART, and VLS. VLS data was shared with the iDARE coach for data collection purposes to track the process of improving health outcomes. Data was then verified by the iDARE coach by examining the registries during site visits.

Program data was collected and maintained on the secured organization drive. The data was never paired with individually identifiable markers, which were not collected by the program teams.

Data will be analyzed using R for descriptive and inferential statistics (version 4.1.2; R Foundation for Statistical Computing).

## Results

Implementation began in September 2020 for iDARE in Tororo District at Nagongera HC IV, followed by Mulanda HC IV in October 2020, then in Kapachorwa District at Kabeywa HC III in February 2021. The baseline was assessed the preceding month before implementation at each of the sites to determine the total number of men and children classified as active in care and the total number of those that were active in care that had achieved VLS. The average across all sites at baseline indicated 72% (668/992) of males and 63% (103/163) of children active in care had achieved VLS. Baseline data across all 3 pilot sites are provided in Table 1 below.

**Table 1:** Cohorts by group and facility at baseline.

Cohorts			Nagongera HCIV (August 2020)		Mulanda HCIV (September 2020)		Kabeywa HCIII (January 2021)		Total
**Children (male and female, 0-19 years)**									
	Virally suppressed, n (%)	62 (60%)		38 (73%)		3 (38%)		103 (63%)	
	Active in Care, n	103		52		8		163	
**Men (20 years and over)**									
	Virally Suppressed, n (%)	357 (65%)		296 (85%)		15 (60%)		668 (72%)	
	Active in Care, n	550		347		25		922	

## Discussion

Data on VLS was collected from facilities on a monthly basis to record progress towards the 95-95-95 goal. The expected primary outcome was increasing actively enrolled men and children reaching VLS to meet the Ugandan MOH’s target of 95% viral suppression among those active in care. Solutions identified as successful are to be shared as an evidence base for scale up at the national level through leveraging information sharing through the social and behavior change packages developed over the course of implementation. Due to the high level of contextualization of solutions, social and behavior change packages will need to be continually updated to track lessons learned, highlight best practices, and include up-to-date data on the ongoing implementation of iDARE to address GYSI gaps in HIV care, including in VLS.

### Conclusions

The data collected on the iDARE implementation as a means to increase VLS among men and children will aid in the contribution to the evidence base of addressing GYSI issues impacting access and uptake of services. Additionally, anticipated results will reflect the necessity of using locally-led, inclusive quality improvement techniques that GYSI based barriers to care, such as iDARE, are a key element in the global goal to end the HIV pandemic.

Limitations in the design include the nonexperimental comparison. To support Uganda’s goal to close the gap in achieving the HIV 95-95-95 goals, next step recommendations include comparison site data on VLS for men and children as a means to determine a more accurate effect size for iDARE.

## Abbreviations

ART: antiretroviral therapy
CCP: Johns Hopkins Center for Communications Program
GYSI: gender, youth, and social inclusion
HC: health center
HIVDR: HIV antiretroviral drug resistance
MOH: ministry of health
SBC: social and behavior change
SBCA: Social and Behavior Change Activity
SSA: Sub-Saharan Africa
USAID: United States Agency for International Development
VLS: viral load suppression
